# Impact of menopause on the mental health of women in the basic health center of Réjiche, Tunisia

**DOI:** 10.1192/j.eurpsy.2023.2396

**Published:** 2023-07-19

**Authors:** F. Zaouali, A. Ben Slama

**Affiliations:** Health Care Center of Réjiche, Basic health group of Mahdia, Mahdia, Tunisia

## Abstract

**Introduction:**

Menopause is a special period for women which can have both physical and psychological consequences.

**Objectives:**

The aim of our study was to assess the impact of menopause on women’s quality of life.

**Methods:**

A cross-sectional descriptive study conducted on menopausal women consulting at the basic health center in réjiche over a period from 12 september to 12 october 2022. The impact of menopause on the mental health of women was assessed by the menopause rating scale (MRS).

**Results:**

A total of 83 women were incluted in our study. The mean age was 61.89±11.03 years. The median age of menopause was 45 years (50-43). The majority of women (90.4%) were married and five women (6%) lived alone. Twenty-six women were professionally active and more than half were sedentary (54.2%). The comorbidities were dominated by arterial hypertension (50.6%). The mean BMI was 30.6±4.75 Kg/m². All the women had a variable psychological impact (from minor to very strong). The median score of the psychological scale was 10 (13-7): median scores for depressed mood, anxiety and physical and intellectual fatigue equal to 3 (4-1), each and median score for irritability equal to 2 (4-1). Physical and mental fatigue was the most common psychological symptom in 88% of cases followed by irritability in 86.7% of patients. Depressive and anxious symptoms were noted in 85.5% and 84.3% of cases, respectively. Psychological impact was severe in 33.7% of patients (strong or very strong symptoms).

**Conclusions:**

Focusing on a small sample of menopausal patients, our study revealed a high prevalence of psychological distress during menopause which requires early adequate assessment and treatment.

**Disclosure of Interest:**

None Declared

